# Effect of Postnatal Nutritional Environment Due to Maternal Diabetes on Beta Cell Mass Programming and Glucose Intolerance Risk in Male and Female Offspring

**DOI:** 10.3390/biom11020179

**Published:** 2021-01-28

**Authors:** Danièle Bailbe, Junjun Liu, Pengfei Gong, Bernard Portha

**Affiliations:** 1 Laboratoire B2PE (Biologie et Pathologie du Pancréas Endocrine), Unité BFA (Biologie Fonctionnelle et Adaptive), Equipe 1, Université de Paris, UMR8251, CNRS, F-75013 Paris, France; danielle.bailbe@univ-paris-diderot.fr (D.B.); ftdxfish@yahoo.fr (J.L.); hbugpf@163.com (P.G.); 2 Shandong Institute of Endocrine & Metabolic Diseases, Shandong First Medical University & Shandong Academy of Medical Sciences, Jinan 250062, China

**Keywords:** postnatal nutrition, beta cell mass, diabetes risk, maternal diabetes, endocrine programming, embryo transfer, GK rat

## Abstract

Besides the fetal period, the suckling period is a critical time window in determining long-term metabolic health. We undertook the present study to elucidate the impact of a diabetic suckling environment alone or associated with an in utero diabetic environment on beta cell mass development and the risk of diabetes in the offspring in the long term. To that end, we have compared two experimental settings. In setting 1, we used Wistar (W) rat newborns resulting from W ovocytes (oW) transferred into diabetic GK rat mothers (pGK). These oW/pGK neonates were then suckled by diabetic GK foster mothers (oW/pGK/sGK model) and compared to oW/pW neonates suckled by normal W foster mothers (oW/pW/sW model). In setting 2, normal W rat newborns were suckled by diabetic GK rat foster mothers (nW/sGK model) or normal W foster mothers (nW/sW model). Our data revealed that the extent of metabolic disorders in term of glucose intolerance and beta cell mass are similar between rats which have been exposed to maternal diabetes both pre- and postnatally (oW/pGK/sGK model) and those which have been exposed only during postnatal life (nW/sW model). In other words, being nurtured by diabetic GK mothers from birth to weaning was sufficient to significantly alter the beta cell mass, glucose-induced insulin secretion and glucose homeostasis of offspring. No synergistic deleterious effects of pre-and postnatal exposure was observed in our setting.

## 1. Introduction

Type 2 diabetes (T2D), of which the prevalence worldwide has reached pandemic proportions, is a complex polygenic disease that often manifests years before its eventual clinical diagnosis [1]. Abnormalities in beta cell function are critical in defining the T2D risk because T2D installs only when beta cell function deteriorates and fails to compensate for insulin resistance in peripheral tissues [2]. T2D is believed to be the result of complex interactions between an individual’s genome and environmental cues. Although single nucleotide polymorphisms at multiple genetic loci have been shown to be associated with T2D, for the majority of people with T2D, only a small proportion (5% to 10%) can be explained by genetic background [3]. Environmental factors such as consumption of a high-fat, high-sugar diet and/or inactivity in adults and their interaction with the genome are classically thought to be critical in the determination of T2D risk [4,5]. The fetal and early postnatal environments are also considered to be crucial in shaping long-term health [6], and a substantial body of evidence links parental diabetic status to metabolic traits in offspring. Extensive epidemiological and experimental evidence has shown that exposure to an adverse intrauterine environment, as observed in the offspring of pregnancies complicated by diabetes, can program susceptibility to metabolic and endocrine disorders in later life [7,8,9]. Disturbances in the development of the endocrine pancreas have been observed when the availability of nutrients was increased or decreased. In rats, intrauterine metabolic perturbations induced by several means such as manipulation of the maternal diet, e.g., protein or calorie restriction, or alteration in the availability of nutrients by placental insufficiency or maternal diabetes alter the islet development at the perinatal period and provoke lasting consequences. Besides the fetal period, the suckling period has also been shown to be a critical time window in determining long-term metabolic health. A large number of animal studies employing early weaning, cross-fostering or nutritional manipulation targeting the lactation period only [10] support the notion that the early postnatal period is critical for energy metabolism disturbances to develop in the offspring.

The existing models to study the influence of a specific period, especially the fetal period, although useful, have drawbacks (see the discussion in [8]). To circumvent the multiple limitations, we proposed that embryo transfer experiments might represent a more relevant paradigm [11].

We undertook the present study to discriminate between the impact of combined in utero and postnatal maternal diabetes and the postnatal diabetic environment alone on beta cell mass development and the risk of glucose metabolism alteration in offspring in the long term. This was achieved in two different experimental settings. In setting 1, based on an embryo transfer approach [11], we used Wistar (W) rat newborns obtained from W ovocytes (oW) and transferred them into diabetic GK rat mothers (pGK). These oW/pGK neonates were then suckled by diabetic GK foster mothers (oW/pGK/sGK model). In setting 2, in order to identify the proper contribution of the isolated postnatal diabetic environment, we proceeded to cross-foster normal W rat newborns (with normal beta cell mass at birth) to diabetic GK rat foster mothers or normal non-diabetic W mothers. Finally, at variance with many postnatal nutritional animal studies focused on male offspring, we investigated potential gender differences in beta cell and glucose metabolism adaptations in response to postnatal exposure to maternal diabetes.

## 2. Materials and Methods

### 2.1. Animals

All animal experimentation was conducted on GK/Par (GK) rats and non-diabetic Wistar (W) rats from our local colonies maintained at the University Paris-Diderot animal core and in accordance with the accepted standards of animal care laid out in the French National Center for Scientific Research 2010 guidelines. The characteristics of the adult GK rats have been described previously [12].

### 2.2. Embryo Harvesting and Transfer Procedures

W embryos were obtained from donor female W rats caged in groups of up to four with a male of proven fertility (E0) and allowed to mate overnight. The following morning, coitus was confirmed by the presence of a vaginal plug, and embryos were harvested in the early afternoon at E0.5 (one-cell stage). All embryos were recovered and transplanted surgically into recipient females as described by Cozzi et al. [13]. The success of W ovocyte transfer in 18 GK females, measured as the ratio of the total number of living newborns to the total number of transferred ovocytes, was 36% (18 transferred females, 206 transferred ovocytes, 15 pregnant females, 60 living newborns). This has to be compared with the 65% value obtained for W ovocyte transfer in 20 W females (10 transferred females, 105 transferred ovocytes, 8 pregnant females, 60 living newborns). Litter size at birth averaged 5.6 in the oW/pGK group and averaged 5.7 in the oW/pW group. Duration of gestation was not modified by the ovocyte transfer protocol in either group as compared to mean values (22 days) observed in the W stock groups. Each litter size was culled to 6 to 8 pups.

### 2.3. First Set of Experiments: Embryo-Transferred and Cross-Fostered Groups

**Figure 1:**
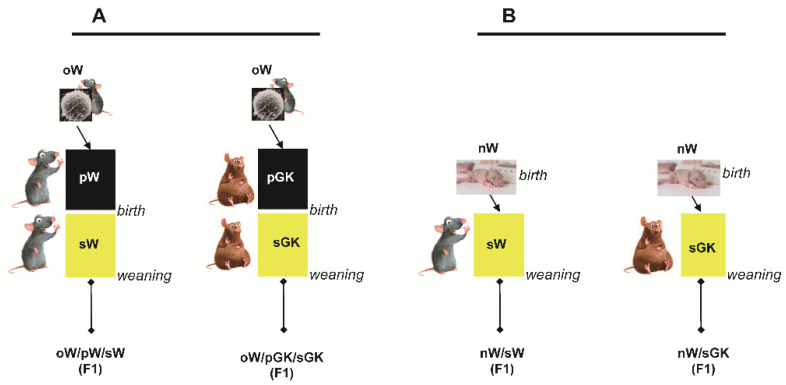
Flow chart illustrating the F1 rat groups reared under control (oW/pW/sW and nW/sW) and exposure to maternal diabetes (oW/pGK/sGK and nW/sGK) conditions. (**A**): Adult animals were obtained from Wistar (W) ovocytes (oW), transferred into W mothers (pW) and reared with W dams (oW/pW/sW group) or obtained from oW ovocytes, transferred into GK mothers and reared with GK dams (oW/pGK/sGK group). (**B**): Adult animals were issued from W neonates and suckled by GK foster mothers (nW/sGK group) or W foster mothers (nW/sW group). oW: Wistar ovocyte; pGK: pregnant GK mother; pW: pregnant W mother; sGK: suckling GK mother; sW: suckling W mother.

Several experimental conditions were designed (Figure 1). Ovocytes obtained from W rats were transferred into pseudo-pregnant GK females. The newborns delivered by the GK mothers were then suckled by GK foster mothers (sGK) (oW/pGK/sGK offspring). The control group consisted of ovocytes obtained from W rats and transferred back into pseudo-pregnant W female rats. The newborns delivered by the W mothers were then suckled by W foster mothers (oW/pW/sW offspring). The last group consisted of non-manipulated W stock newborns raised in parallel. Both male and females were studied.

### 2.4. Second Set of Experiments: Cross-Fostered Groups

W newborns (nW) delivered by W mothers were suckled by GK foster mothers (sGK) or W foster mothers (sW). The nW/pGK group was compared to the nW/pW group (Figure 1). In each group, both males and females were studied sequentially at 4 and 8 weeks of age.

### 2.5. Glucose Tolerance and Insulin Secretion Tests

In the first step of experiments, IVGTTs (0.5g glucose/kg body weight) were performed under pentobarbital sodium anesthesia (80 μl/100 g body weight I.P.; Ceva Santé Animal, Libourne, France) in 10-week-old rats, as previously described [14]. Blood samples were collected sequentially from the tail vein before and 5, 10, 15, 20, 30 and 60 min after the injection of glucose. In the second step of experiments, IPGTTs (1 g glucose/kg body weight) were performed in freely moving 4- and 8-week-old rats, as previously described [14]. Blood samples were collected sequentially from the tail vein before and 15, 30 and 60 min after glucose administration. Plasma glucose and insulin were determined at each time point, as previously described [14].

At the end of the GTTs, pancreases were excised and weighed. For determination of insulin content, pancreases were homogenized and centrifuged (1500× *g*) at 4 °C in an acid/alcohol solution (75% ethanol, 1.5% 32.5 mol/L HCl and 23.5% distilled water). The supernatant fraction was stored at −20 °C until insulin was assayed. For endocrine cell immunohistochemistry and morphometry, pancreases were fixed in aqueous Bouin solution and embedded in paraplast according to standard procedures [11].

### 2.6. Immunohistochemical Analysis

Each pancreatic block was serially sectioned (5 µm) throughout its length and was then mounted on slides. Sections at fixed intervals throughout the block (every 35th section) were immunostained for insulin using a technique adapted from peroxidase indirect labeling, as previously described [14,15].

### 2.7. Morphometric Image Analysis

Quantitative beta cell evaluation was performed with an Olympus BX40 microscope equipped with a color video camera and using the computer-assisted image analysis system Histolab 5.2 software (Microvision Instruments, Evry, France), as previously described [14,15]. The area of insulin-positive cells and that of total pancreatic sections were evaluated in each stained section. Beta cell relative volume was determined according to stereological methods by calculating the ratio between the area occupied by insulin immunoreactive cells and that occupied by total pancreatic cells. Total beta cell mass per pancreas was derived by multiplying the total pancreatic weight by the beta cell relative volume. At least six sections were analyzed for each pancreas. Six rats were analyzed per experimental group.

### 2.8. Calculations

The insulin and glucose responses during the GTTs were calculated as the incremental plasma insulin values integrated over a period of 60 min after the administration of glucose (Δ*I*, pmol·L^−1^·min^−1^) and the corresponding increase in glucose concentration (Δ*G*, mmol·L^−1^·min^−1^), respectively. The insulinogenic index (Δ*I*/Δ*G*) represents the ratio of these two variables. All results are expressed as mean ± SEM, with the number of observations and significance between group differences evaluated by one-way ANOVA followed by Tukey–Kramer post hoc tests. A *p* value < 0.05 was considered significant.

## 3. Results

### 3.1. Diabetes Phenotype in Pregnant and Lactating GK Females

**Figure 2:**
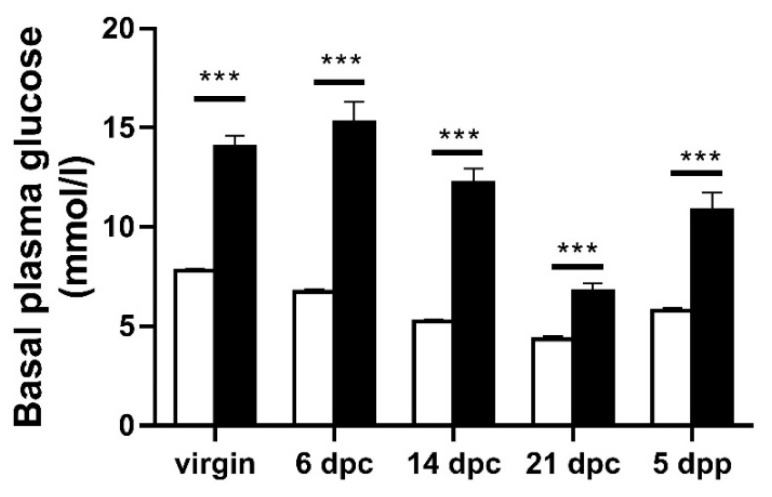
Basal plasma glucose level (post-absorptive state) in GK (black bars) and Wistar rat (white bars) females before pregnancy (virgin), during pregnancy on days 6, 14 and 21 (day post-coitum, dpc) and during lactation on day 5 after delivery (day postpartum, dpp). Duration of pregnancy was the same in both groups (21.5 days). Values are mean ± SEM. Nine to twelve rats were analyzed per experimental group. *** *p* < 0.001 as compared to age-related value in the W group.

When compared to W pregnant controls, the GK females exhibited a higher plasma glucose level throughout the gestation and lactation periods (Figure 2). These data demonstrate that the pregnant and lactating GK female can be viewed as a spontaneous and reproducible model of mild and stable diabetes that allows us to look at the consequences of diabetes during lactation to the offspring.

### 3.2. Adult W/pGK/sGK Offspring Have Decreased Beta Cell Mass and Low Glucose-Stimulated Insulin Secretion and They Are Glucose Intolerant

To assess the effects of combined in utero and postnatal maternal diabetes on glucose homeostasis in the oW/pGK/sGK model, we studied adult offspring from six litters of rats issued from W ovocyte transfer into GK females and suckled by GK foster mothers (oW/pGK/sGK). Comparison was made with values in the oW/pW/sW and W stock animals when indicated.

The 10-wk-old oW/pGK/sGK male rats had a mean body weight similar to that of the oW/pW/sW males (Table 1). As compared to the oW/pW/sW group, the 10-wk-old oW/pGK/sGK males exhibited normal post-absorptive plasma glucose levels, low pancreatic insulin content and low beta cell mass (*p* < 0.001 and *p* < 0.02, respectively) (Table 1). Similarly, the females from the oW/pGK/sGK group exhibited lower pancreatic insulin content compared to the oW/pW/sW females.

**Figure 3:**
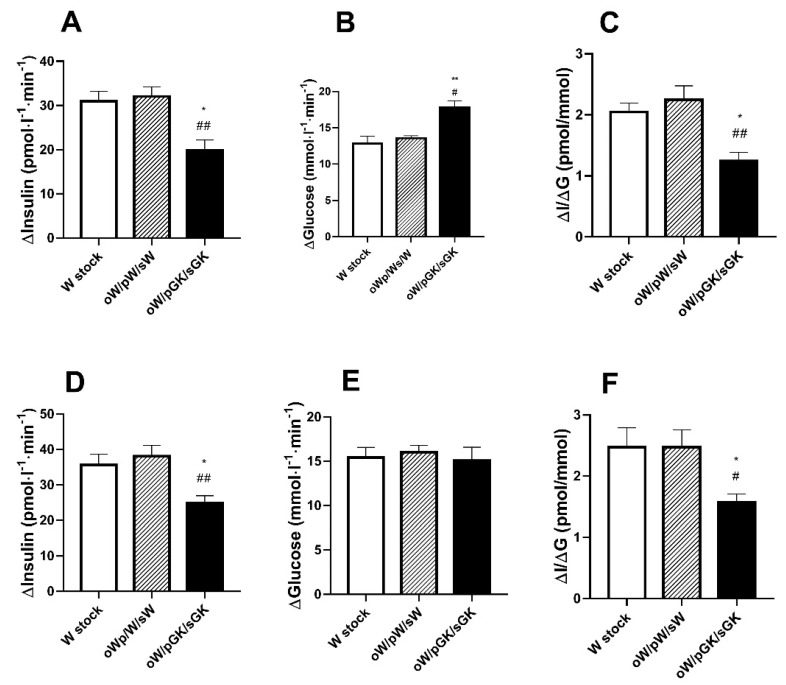
Glucose tolerance (Δ*G*), glucose-stimulated insulin secretion (Δ*I*) and insulinogenic index (Δ*I*/Δ*G*) in 10-wk-old male (**A**–**C**) or female (**D**–**F**) rats. Animals were obtained from W ovocytes (oW), transferred into W mothers (pW) and reared with W dams (oW/pW/sW group) or obtained from oW ovocytes, transferred into GK mothers and reared with GK dams (oW/pGK/sGK group). They were compared to control W rats (W stock, no transfer). oW: Wistar ovocyte; pGK: pregnant GK mother; pW: pregnant W mother; sGK: suckling GK mother; sW: suckling W mother. In vivo glucose tolerance and insulin secretion were tested in response to an intravenous (i.v.) injection of glucose (0.5 g/kg body weight) in anesthetized rats (post-absorptive state). Values are mean ± SEM. Nine to twelve rats were analyzed per experimental group. * *p* < 0.05; ** *p* < 0.01; as compared to value in sex-related oW/pW/sW group. # *p* < 0.05; ## *p* < 0.01 as compared to value in in sex-related W stock group.

In males, after glucose injection, the AUC glucose (Δ*G*) raised to levels markedly higher than those in stock W males (*p* < 0.01) and in oW/pW/sW (*p* < 0.05) (Figure 3), whereas in females, the AUC/glucose was similar between the three groups. In response to glucose, in both males and females, the AUC/insulin (Δ*I*) levels were markedly lower than those in the stock W (*p* < 0.001) and the oW/pW/sW rats (*p* < 0.01) (Figure 3). Accordingly, their mean insulinogenic index (Δ*I*/Δ*G*) was low as compared to that of the stock W (*p* < 0.05) and the oW/pW/sW rats (*p* < 0.01) (Figure 3).

### 3.3. Adult nW/sGK Offspring Have Decreased Beta Cell Mass and Low Glucose-Stimulated Insulin Secretion and They Are Glucose Tolerant

To assess any possible long-term effect of the diabetic postnatal suckling environment alone (from birth to weaning) on glucose homeostasis, we studied adult offspring from six litters of Wistar newborns (nW) issued from W mothers and suckled by GK foster mothers (nW/sGK).

**Figure 4:**
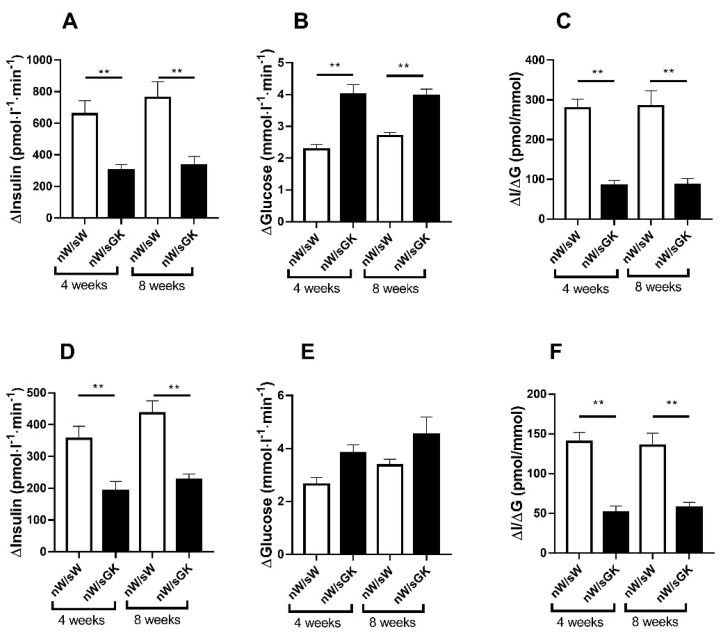
Glucose tolerance (Δ*G*), glucose-stimulated insulin secretion (Δ*I*) and insulinogenic index (Δ*I*/Δ*G*) in 4- and 8-wk-old male (**A**–**C**) or female (**D**–**F**) rats. Animals were issued from W neonates suckled by GK foster mothers (nW/sGK group) or issued from W neonates suckled by W foster mothers (nW/sW group). In vivo glucose tolerance and insulin secretion were tested in response to an i.p. injection of glucose (1 g/kg body weight) in freely moving rats (post-absorptive state). Values are mean±SEM. Nine to thirteen rats were analyzed per experimental group. ** *p* < 0.01 as compared to value in the age- and sex-related nW/sW group.

At 4 (just before weaning) and 8 weeks of age, a comparison was made with phenotype in an nW/sW group raised in parallel. The 4-wk-old nW/sGK males weighed significantly less (*p* < 0.01) than the nW/sW males (Table 2). As compared to the nW/sW group, the 4-wk-old nW/sGK males exhibited normal basal plasma glucose levels, lower pancreatic insulin content and lower beta cell mass (*p* < 0.01) (Table 2). After glucose administration, their plasma glucose levels and AUC/glucose (Δ*G*) raised to levels markedly higher (*p* < 0.01) than those in nW/sW males (Figure 4). In response to glucose, their AUC/insulin (Δ*I*) and their mean insulinogenic index (Δ*I*/Δ*G*) were markedly lower (*p* < 0.01) than those in the nW/sW males (Figure 4).

**Figure 5:**
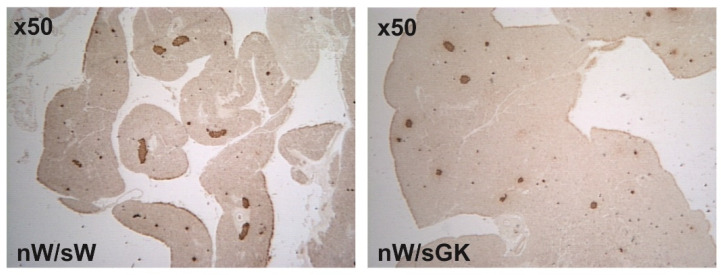
Representative immunoperoxidase staining for pancreatic insulin in 8-wk-old male rats. Animals were issued from W neonates suckled by GK foster mothers (nW/sGK group), or issued from W neonates suckled by W foster mothers (nW/sW group).

At 8 weeks of age, nW/sGK males had returned to normal body weight. As compared to the nW/sW group, the 8-wk-old nW/sGK males still exhibited normal basal plasma glucose levels, low pancreatic insulin content and low beta cell mass (*p* < 0.01) (Table 2 and Figure 5).

After glucose administration, their plasma glucose levels and AUC/glucose (Δ*I*/Δ*G*) raised to levels markedly higher (*p* < 0.01) than those in nW/sW males (Figure 4). In response to glucose, their AUC/insulin (Δ*I*) and their mean insulinogenic index (Δ*I*/Δ*G*) were markedly lower (*p* < 0.01) than those in the nW/sW males (Figure 4).

A similar pattern was found in 4- and 8-wk-old nW/sGK females raised in parallel, with a mean Δ*I* insulin and a mean insulinogenic index (Δ*I*/Δ*G*) markedly lower (*p* < 0.01) than those in the nW/sW females (Figure 4).

From the data obtained here, we concluded that suckling of a non-diabetic W offspring by a diabetic GK mother alone is sufficient to significantly increase the risk of intolerance to glucose at adult age.

## 4. Discussion

In the current study, we sought to dissect the impact of a diabetic postnatal nutritional environment, either alone or in combination with an in utero diabetic environment, on the metabolic phenotype of offspring in adulthood. We followed the phenotype of Wistar rats who were cross-fostered from birth to weaning onto diabetic GK dams (nW/sGK model). At 4 weeks of age (just before weaning), the nW/sGK males exhibited low body weight, low pancreatic insulin content and low beta cell mass. This was associated with low glucose-induced insulin secretion and glucose intolerance, as compared to the normal phenotype (nW/sW model). At 8 weeks of age (young adult), the nW/sGK males had regained normal body weight, but they still suffered low pancreatic insulin content, low beta cell mass, low glucose-induced insulin secretion and glucose intolerance. One may conclude that non-diabetic W offspring suckling by a diabetic mother from birth to weaning adversely and irreversibly impacts beta cell mass and is associated with long-term impairment of insulin secretion and glucose tolerance. This conclusion supports the notion first proposed by Patel and Srinivasan [16] that metabolic programming of the endocrine pancreas may occur during the neonatal nutrition period, with their demonstration that artificially rearing rat pups on high-carbohydrate milk during the suckling period induces glucose intolerance and impaired insulin secretion [16,17].

It is clear that an altered growth profile in the postnatal period can affect adult metabolic homeostasis [18,19,20]. This is a matter of concern in the nW/sGK model since we observed growth retardation until weaning in W pups reared by GK mothers compared with W pups reared by non-diabetic W mothers (nW/sW rats). Increased glucose levels as well as decreased milk synthesis and milk ejection have been reported in lactating streptozotocin-induced diabetic rats [21]. If this also applies to GK mothers, it may lead to both qualitative and quantitative alterations in nutrition, culminating in an undernutrition-like state of the nW/sGK pups. It has long been appreciated that neonatal growth retardation is associated with increased risk in development of T2D [22,23,24]. Could a potential postnatal nutrient restriction be the major contributor for acquisition of the nW/sGK rat phenotype? Previous investigations employing caloric restriction (by 50%) during the suckling phase in rats revealed virgin female offspring that are lean and glucose tolerant, despite a reduced ability to secrete insulin in response to a glucose challenge [25]. Selective postnatal nutrient restriction also influenced beta cell mass development. Postnatal caloric restriction (by 50%) resulted in decreased pancreatic weight. However, beta cell fractional area and beta cell mass (adjusted for body weight) were unexpectedly increased compared with the control [26]. Most notably, when superimposed on a rat model of intrauterine growth restriction, postnatal nutrient restriction was found to be protective against obesity, hepatic insulin resistance and glucose intolerance [25]. Isolated postnatal growth restriction accomplished by nutrient restriction, whether protein or calories, imposed during the suckling phase only, is metabolically protective in rodent offspring [25]. This pattern of rat models with isolated postnatal growth restriction is not convergent with that observed in the nW/sGK rat model. To partially explain this discrepancy, we propose an argument which applies specifically to the GK model. Indeed, in the GK model, other factors such as maternal behavior (licking and grooming behavior, nest construction) are altered (Darnaudery M., Movassat J. and Portha B. unpublished observation) [27]. This important defect, which was not present in the other models of growth restriction cited above, may significantly contribute to the outcome.

Finally, we also observed a significant influence of offspring sex on the development of the phenotypes. In contrast to the male offspring of diabetic dams who developed severe metabolic changes, findings in the female offspring were mild. Some authors have suggested a protective effect of estrogen for metabolic traits in females [28,29,30], a hypothesis that is not well understood in a perinatal programming model and needs to be investigated further.

Since suckling of a W offspring (with no predisposition to develop diabetes) by a diabetic mother negatively impacts the programming of its beta cell mass and is associated to long-term glucose intolerance, we then wanted to know whether the combination of postnatal maternal environment with in utero exposure to maternal diabetes is more detrimental for the metabolic health of the progeny than sole postnatal suckling by diabetic mothers. To our surprise, our present data obtained with the oW/pGK/sGK model revealed that the extent of metabolic disorders in terms of glucose intolerance and beta cell mass are similar between rats which have been exposed to maternal diabetes both pre- and postnatally and those which have been exposed only during postnatal life. In other words, being nurtured by diabetic GK mothers from birth to weaning was sufficient to significantly alter the glucose homeostasis of offspring, and no synergistic deleterious effects of pre- and postnatal exposure were observed in our setting.

In our study, the majority of the defects were present in both sexes, except for glucose intolerance. Indeed, the young male oW/pGK/sGK offspring maintained normal basal blood glucose, but they developed glucose intolerance, whereas young female oW/pGK/sGK offspring were less susceptible and kept normal tolerance to glucose.

Interestingly, the impact of postnatal feeding by a diabetic mother on beta cell mass development appears quite different from that of postnatal nutrient restriction. Garofano et al. [31] and Matveyenko et al. [26] have independently reported that beta cell mass was reduced by 40–50% in 21-day-old pups exposed to intrauterine caloric restriction followed by exposure to normal nutrient availability and by 70–80% in rats exposed to both intrauterine and postnatal caloric restriction. This indicates, in this case, that superimposition of postnatal nutrient restriction amplifies the effect of a previous intrauterine restriction, while superimposition of postnatal feeding by a diabetic mother does not aggravate the potential effect of a previous prenatal exposure to maternal diabetes.

Our understanding of the mechanisms underlying reduced beta cell mass in response to maternal diabetes in particular, and to inappropriate perinatal nutrition in a more general perspective, is limited so far. The relative contributions of the many intrinsic and extrinsic factors which contribute to the plasticity of the developing endocrine pancreas are yet to be established. The most promising perspective relies on identification of epigenetic modifications affecting the expression of key genes critical for endocrine pancreas development and beta cell function in the offspring of various models of metabolic abnormalities during pregnancy, including diabetes [32,33]. This epigenomic information has been correlated with changes in offspring pancreas and proposed as one mechanism that could contribute to the development of later-life metabolic disorders (see detailed review in [9,34]).

In summary, postnatal suckling by a diabetic mother from birth to weaning in both male and female rats led to an inappropriate postnatal development of beta cell mass. Importantly, nursing non-diabetes-prone pups by diabetic mothers was revealed to be a critical factor with major endocrine and metabolic influences, since the acquired low beta cell mass phenotype is maintained on the long-term and it is associated with glucose intolerance in the adults. In contrast, in male and female rats previously exposed to prenatal maternal diabetes/hyperglycemia, superimposition of nursing by diabetic mothers did not aggravate the low beta cell mass phenotype (Movassat and Portha unpublished results [35]). Therefore, although introduction of postnatal feeding by a diabetic mother in a normal rat neonate has long-term negative implications, these effects are not cumulative in the case of combination of in utero and postnatal exposure to a diabetic environment. We are currently addressing the question of sole in utero exposure to maternal diabetes using an embryo transfer protocol in which oW/pG rats are fostered by non-diabetic Wister dams. In this model, the ovocyte enter pregnancy in a euglycemic state, will be exposed to hyperglycemia throughout the pregnancy and return postpartum to a normoglycemic environment.

To conclude, the nW/sGK and oW/pGK/sGK rat models used in the present study represent new paradigms optimized for identifying the potential mechanisms of beta cell dysfunction programmed by maternal diabetes. They allow for deciphering the exact timing in perinatal life at which diabetes is most impressionable on the developmental programming of the endocrine pancreas and, more generally, of metabolically relevant tissues. These models offer perspective to develop interventions that can rescue beta cell programming. For example, nutrient restriction during the last week of gestation in GK mothers increases beta cell mass in their fetuses [35], and neonatal treatment with glucagon-like peptide 1 receptor agonists prevents the loss of beta cells in intrauterine growth restricted pups [36] and showed efficient amelioration of the adult phenotype (improved beta cell mass and milder diabetes) in diabetes-prone GK pups [37]. Finally, our work supports the hypothesis that pre- and postnatal maldevelopment of islets, leading to insufficient insulin secretion, may be an important, yet underappreciated, risk for the subsequent manifestation of type 2 diabetes [38,39]. Accordingly, the deficit in beta cell mass found in patients with type 2 diabetes may not only be secondary to an increased rate of apoptosis, as currently considered, but may also be the consequence of abnormal islet cell development and growth.

## Figures and Tables

**Table 1:** Changes in body weight, basal plasma glucose level (post-absorptive state), pancreatic insulin stores and pancreatic beta cell mass in 10-wk-old oW/pW/sW rats obtained from W ovocyte transfer into W mothers and reared with W dams and in 10-wk-old oW/pGK/sGK rats obtained from W ovocyte transfer into GK mothers and reared with GK dam. oW: Wistar ovocyte; pGK: pregnant GK mother; pW: pregnant W mother; sGK: suckling GK mother; sW: suckling W mother.

Rats	Sex	Body Weight (g)	Basal Plasma Glucose (mmol/L)	Pancreatic Insulin Stores (pmol/mg pancreas)	Pancreatic Beta Cell (%)	Beta Cell Mass (μg/mg pancreas)
oW/pW/sW	Male	362 ± 18 (10)	6.6 ± 0.2 (10)	23.8 ± 1.0 (10)	0.51 ± 0.03 (6)	5.10 ± 0.30 (6)
	Female	246 ± 5 (11)	6.4 ± 0.1 (11)	25.3 ± 0.5 (11)	ND	ND
oW/pGK/sGK	Male	338 ± 10 (11)	6.0 ± 0.1 (11)	16.4 ± 0.8 ** (14)	0.40 ± 0.03 * (6)	3.98 ± 0.25 * (6)
	Female	225 ± 5 (19)	5.9 ± 0.1 (19)	16.3 ± 0.5 *** (11)	ND	ND

* *p* < 0.02; ** *p* < 0.01; *** *p* < 0.001 as compared to related value in corresponding male or female oW/pW/sW group. ND: not determined. (): number of individuals in each group.

**Table 2:** Changes in body weight, basal plasma glucose level (post-absorptive state), pancreatic insulin stores and pancreatic beta cell mass in 4- and 8-wk-old male rats issued from W neonates (nW) suckled by W foster mothers (nW/sW group) and in 4- and 8-wk-old male rats issued from W neonates suckled by GK foster mothers (nW/sGK group).

Age	Rats	Body Weight (g)	Basal Plasma Glucose (mmol/L)	Pancreatic Insulin Stores (pmol/mg pancreas)	Pancreatic Beta Cell (%)	Beta Cell Mass (μg/mg pancreas)
4 wks	nW/sW	85 ± 1 (10)	6.7 ± 0.3 (10)	ND	ND	ND
4 wks	nW/sGK	65 ± 3 ** (10)	7.2 ± 0.3 (16)	ND	ND	ND
8 wks	nW/sW	221 ± 10 (9)	7.1 ± 0.2 (9)	28.0 ± 1.0 (6)	0.56 ± 0.03 (6)	5.6 ± 0.3 (6)
8 wks	nW/sGK	212 ± 9 (9)	7.4 ± 0.2 (9)	19.5 ± 1.0 ** (6)	0.43 ± 0.02 ** (6)	4.3 ± 0.2 ** (6)

**, *p* < 0.01 as compared to related value in the age-related nW/sW group. ND: not determined. ( ): number of individuals in each group.

## Data Availability

Some of these data were presented at the 68th American Diabetes Association Meeting, San Francisco, USA, 18–21 June 2008, and at the 44th European Association for the Study of Diabetes Meeting, Rome, Italy, 8–11 September 2008.
